# Metabolic Syndrome and Psychological Effects of Exercise in Hemodialysis Patients

**DOI:** 10.3390/ijerph182211952

**Published:** 2021-11-14

**Authors:** Beata Hornik, Jan Duława, Jacek Durmała

**Affiliations:** 1 Department of Internal Nursing, School of Health Sciences in Katowice, Medical University of Silesia, 40-752 Katowice, Poland; 2 Department of Internal Medicine and Metabolic Diseases, School of Health Sciences in Katowice, Medical University of Silesia, 40-752 Katowice, Poland; jdulawa@sum.edu.pl; 3 Diaverum, 40-635 Katowice, Poland; 4 Department of Rehabilitation, School of Health Sciences in Katowice, Medical University of Silesia, 40-752 Katowice, Poland; jdurmala@sum.edu.pl

**Keywords:** metabolic syndrome, hemodialysis, physical activity, anxiety, rehabilitation

## Abstract

Metabolic syndrome (MS) and anxiety disorders are common problems among hemodialysis patients (HD). However, there have been no studies defining the role of physical activity in reducing anxiety in HD patients with MS. This study was aimed to determine the effects on the severity of anxiety of a four-week rehabilitation program for HD patients, with or without metabolic syndrome (MS), planned and adapted to their abilities. The study was single-center, interventional, non-randomized, and prospective. Fifty-eight individuals completed the project (28 HD patients and 30 controls (C) with normal kidney function). Each group was divided into two subgroups with respect to MS. The mean age of the subjects in the HD and C groups was 56.9 ± 13.3 years ($\bar{x}$ ± SD) and 61.5 ± 8.3 years ($\bar{x}$ ± SD), respectively. Planned and adapted to the patient’s abilities, the rehabilitation program based on physiotherapy was provided to each subject for 4 weeks. Baseline and post-intervention determined anxiety levels using the State-Trait Anxiety Inventory (STAI). The X1 scale tests state anxiety, and the X2 scale tests trait anxiety. Post-intervention, there was no significant difference in the intensity of state anxiety observed in HD patients compared to C with normal renal function, as observed before the program. After four weeks of regular physical activity planned and adapted to the patient’s abilities in an inpatient ward, the level of state anxiety (X1) and trait anxiety (X2) lowered considerably in all HD patients (respectively: 35.1 ± 8.0 vs. 29.2 ± 5.0, *p* = 0.001 for X1 and 41.8 ± 9.1 vs. 38.1 ± 5.9, *p* = 0.008 for X2). The rehabilitation program significantly reduced the intensity of state anxiety (X1) in HD patients with MS (35.8 ± 7.9 vs. 29.2 ± 5.1; *p* = 0.01). The rehabilitation program helped to significantly reduce the intensity of trait anxiety (X2) in HD patients without MS (41.9 ± 10.7 vs. 36.9 ± 5.9; *p* = 0.04). Four-week physical activity planned and adapted to the patient’s abilities reduces the intensity of anxiety in HD patients and controls with normal renal function. HD patients with MS benefit more in terms of reducing perceived state anxiety, and HD patients without MS in terms of reducing trait anxiety.

## 1. Introduction

Metabolic syndrome (MS) is a complex disorder characterized by a combination of factors. Each of those factors predisposes an individual to cardiovascular risk. The most frequently used diagnosis criteria are those proposed by the World Health Organization (WHO), the International Diabetes Federation (IDF), and the National Cholesterol Education Program—Adult Treatment Panel (NCEP ATP) [1].

According to various studies, MS occurs in about 50–75% of hemodialysis (HD) patients [2,3]. The estimates of the comorbidity of anxiety or depression and MS are also ambiguous [4,5,6]. Some authors indicate a relationship between MS and depression [7,8]. Others reveal only the relationship between MS and anxiety [9,10].

Anxiety disorders are the most common mental health problem in the world. About eight percent of the population experience them once a year. Fourteen percent endure them at least once in their lives [11].

The prevalence of anxiety of varying severity in HD patients ranged from 12% to 52% in various studies [12,13,14,15,16,17]. An assessment of depression and suicide risk is always recommended whenever an anxiety disorder is diagnosed [18]. The source of anxiety in chronically HD patients is their current health status and the awareness of reduced control over their own lives. In the United States, withdrawal from dialysis treatment is the third leading cause of death in chronic kidney disease [19]. Moreover, studies show a causal relationship between anxiety and cardiovascular diseases [20,21,22]. Increased anxiety symptoms have also been demonstrated in patients after cardiac incidents. They are related to a twofold increase in the risk of death in patients after coronary artery bypass graft and outpatient patients with coronary artery disease [20,23,24,25].

Physical activity is a recognized non-pharmacological method of reducing anxiety-depressive symptoms [26,27,28]. The beneficial effect of increased physical activity on reducing anxiety in healthy people has been well documented [29,30,31,32]. Conversely, lack of physical activity has been identified as a risk factor for the development of anxiety [33]. However, few studies determine the impact of physical activity on experiencing anxiety in HD patients [34,35,36]. Most of the studies in these patients focused on the beneficial effect of physical activity on reducing depression symptoms [37,38,39]. It seems, therefore, essential to develop the most effective training models to improve the mental and functional condition of dialysis patients [40].

An additional argument for researching the impact of physical activity on anxiety disorders, including the role of metabolic syndrome, is the phenomenon of reversed epidemiology observed in uremic patients [41,42]. Numerous reports on reverse epidemiology deal with the components of MS. The findings that hyperlipidemia and above-normal body weight may be protective could indicate that general rules of epidemiology may not apply to uremic patients [43,44]. However, Lu et al. point out that these factors are no longer protective in patients over 65 [45].

Although there is evidence of the effect of physical activity on treating anxiety [46,47,48], it has not thus far been the subject of research in the context of MS in HD patients. Such an undertaking could provide new information.

Considering the presented facts, it seemed warranted to determine the effect of physical activity on experiencing anxiety depending on the presence or absence of MS. It has been hypothesized that an organized rehabilitation program inhibits fear and anxiety and that the effect may vary depending on the presence of MS. This study was aimed to determine the effects on the severity of anxiety of a four-week rehabilitation program for HD patients with or without metabolic syndrome (MS), planned and adapted to their abilities.

## 2. Participants and Methods

A detailed description of patient enrolment and the study methodology was included in our previous paper [49]. Therefore, we present here an abridged description of the study group and measurements.

### 2.1. Study Design

The study was conducted between January 2013 and December 2016. This was a single-center, interventional, non-randomized prospective study. The study was part of a broader study, part of which was presented in a different context [49], but it was primary research during which new data was collected.

### 2.2. Eligibility Criteria

#### 2.2.1. Inclusion Criteria

The criteria for patient inclusion in the study were: age >18 years, stage 5 chronic kidney disease and hemodialysis treatment duration >3 months (for the study group) or normal kidney function (assessed by e GFR, general urinalysis, 24 h albumin excretion, and renal ultrasound results)—for the control group, the ability of the subject to perform low-level intensity exercise and written informed consent for participation in the study.

#### 2.2.2. Exclusion Criteria

The criteria for exclusion from the study were: a systemic rheumatic disease, cancer, conditions preventing/inability to perform low-level intensity exercise (New York Heart Association class IV heart failure, advanced mobility disorders), overt infection within the last three months, immunosuppressive treatment, pregnancy, and refusal to participate in the study.

### 2.3. Participants and Recruitment

All study participants received printed information about the purpose and manner of the study, and those who decided to participate in the study gave their written consent. These subjects were recruited at the dialysis center in Katowice, Poland. The control group consisted of patients with a normal renal function who participated in a four-week outpatient cardiac rehabilitation program. These subjects were recruited at the Department of Rehabilitation at University Hospital (No. 7 SUM, Upper Silesian Medical Center, Katowice, Poland). Those dialysis center patients who met the inclusion criteria were welcome to participate in the study. In total, 80 subjects were invited to participate in the research (40 dialysis patients and 40 with normal kidney function). Ultimately, sixty-two subjects agreed to participate in the study: 31 dialysis patients and 31 subjects with normal kidney function participating in the rehabilitation program. Fifty-eight completed the project (28 dialysis patients and 30 subjects with normal kidney function).

The reasons HD patients discontinued physical activity included repeated stenocardia pain (one patient who was referred to the cardiology department), worsening of depression (one patient), and loss of motivation (one patient). In the control group, one patient was withdrawn because of loss of motivation.

#### 2.3.1. Assessment of Metabolic Syndrome

The study group and the control group were divided into two subgroups. The distribution criterion was the presence or absence of metabolic syndrome (MS). The MS criterion was adopted according to the 2009 common position of the International Diabetes Federation (IDF) in agreement with the National Heart Institute, Lungs and Blood (NHLBI) and the American Heart Association (AHA) [50]. Diagnosis of MS was based on the presence of at least three out of five distinct, clinically relevant cardio-metabolic risk factors for the European population. The components assessed when diagnosing metabolic syndrome are waist circumference, high-density lipoprotein (HDL), fasting serum triglyceride and glucose concentration, and blood pressure. Its prevalence varies depending on the accepted definition and ethnicity.

The patients were divided into the following groups: study group—hemodialysis patients (HD): patients with metabolic syndrome (14 patients) (HD-MS subgroup); patients without metabolic syndrome (14 patients) (HD-nMS subgroup); control group—subjects with normal renal function undergoing cardiac rehabilitation (C): subjects with metabolic syndrome (13 subjects) (C-MS subgroup), subjects without metabolic syndrome (17 subjects) (C-nMS subgroup). These subjects were recruited at the dialysis center in Katowice, Poland. A control group (C) comprised 30 consecutive patients of the rehabilitation day ward These subjects were recruited at the Department of Rehabilitation at University Hospital. Written informed consent was obtained from each subject after the aim of the study, protocol, and risks were presented and explained.

The mean age of the subjects in the HD and C groups was 56.9 ± 13.3 years ($\bar{x}$ ± SD; in the range from 25 to 75 years) and 61.5 ± 8.3 years ($\bar{x}$ ± SD; in the range from 37 to 76 years), respectively. The HD patient group consisted of 16 women (57%) and 12 men (43%). The group of patients with normal renal function consisted of 15 women (50%) and 15 men (50%).

#### 2.3.2. Assessment of Anxiety

We determined each subject’s anxiety level before and after the rehabilitation using the State-Trait Anxiety Inventory (STAI) [51]. The Polish version of STAI published in 2011 by the copyright owner (Psychological Test Laboratory of the Polish Psychological Association, Warsaw 2011, Poland) was used [52]. The structure of STAI measures the distinction between anxiety understood as transient and the situational condition of the individual and anxiety understood as a relatively constant personality trait. The X1 scale tests state anxiety, and the X2 scale tests trait anxiety. Each of them consists of 20 statements.

The statements included in the scale are formulated in such a way that the highest fear is indicated alternately by the number 4 (a direct statement) or the number 1 (an indirect statement). That prevents mechanical responses and minimizes the impact of nodding. All test items that require result transformation are available in the key. After the switch, the numerical values of responses were added up. A raw score was calculated for a given scale ranging from twenty to eighty. A higher value of the result indicates a greater level of fear and anxiety.

All test items that require result transformation are available in the key. After the switch, the numerical values of responses were added up. A raw score was calculated for a given scale ranging from twenty to eighty. A higher value of the result indicates a greater level of fear and anxiety. The STAI-S and STAI-T scales were conducted using the paper and pencil method. They required direct contact between a dialysis nurse and the patient. The results were assessed by a psychologist who did not know who was from the control group and who was from the study group.

### 2.4. Ethics Statement

The study was conducted following the Helsinki Declaration after the Bioethical Committee of the Medical University of Silesia in Katowice approved the project (Resolution No. KNW/0022/KB1/44/II/06/14/17).

### 2.5. Exercise Program

Before qualifying for the exercise program, all participants had a detailed medical history (the course of the disease, the duration of dialysis treatment), a physical examination, and anthropometric measurements were taken. Before planning an exercise program, a multidisciplinary team assessed locomotion, self-service ability, manual dexterity, balance, range of motion in the spine and peripheral joints, physical performance, and cardiovascular risk. Due to the various possibilities, exercises were customized to match individual capabilities. An individualized supervised rehabilitation program based on physiotherapy was provided to each subject for 4 weeks, 6 days a week, 2 h a day. This program included 20–40 min (25 ± 10 min) of aerobic endurance training (AET)—walking, treadmill walking, forward and back walking, and cycling on stationary ergometers. Every time, the intensity of exercise was closed to ventilatory anaerobic threshold (VAT). During all sessions of AET, the rating of perceived exertion based on Borg 6–20 scale was below 13. It is a subjective method of assessing physical capacity [53]. All patients completed at least 90% of the planned training sessions.

This exercise was complemented with balance and coordination exercises, isometric exercises, exercises in part or full unloading, free active exercises, relaxing exercises, and active breathing exercises. The massage technique and various methods of physical therapy were also used. Most individuals received classic local massage and electrotherapy methods—TENS (transcutaneous electrical nerve stimulation) currents. Of the several electromagnetic treatment methods, mainly constant and low-frequency magnetic fields have been used. In addition to kinesiotherapy, massage, and electrotherapy, light therapy was used, including point laser therapy and local ultrasound.

### 2.6. Statistical Methods

Statistical analyses were performed using the Statistica 13.1 program (StatSoft, Inc., Tulsa, OK, USA). The results of quantitative variables are presented as the mean and standard deviation ($\bar{x}$ ± SD) or on the basis of the median. Qualitative variables measured on nominal and ordinal scales (e.g., education) are presented as numbers (*n*) and percentages (%).

The normality of the empirical distribution of quantitative variables was assessed by the Shapiro–Wilk test. The Mann–Whitney U test was used for non-parametric variables. The Student’s paired t-test for pairs of observations (provided that the distribution was normal) was used to compare the differences or the Wilcoxon test with a distribution deviating from the normal. The result was considered significant if *p* < 0.05.

## 3. Results

The most common causes of chronic kidney disease were: glomerulonephritis (25.1%), diabetic kidney disease (21.4%), and hypertensive nephropathy (21.4%). The mean comorbidity index (Comorbidity Index CI) in the HD and C groups was: 4.5 ± 3.6 and 1.03 ± 0.61, respectively (*p* < 0.001).

HD patients were treated with repeated hemodialysis for 50.6 ± 73.4 months (5 to 272 months). Vascular access was provided by arterio-venous (AV) fistula formation in 24 patients (85.7%), in the remaining patients—by implantation of a synthetic AV graft or using a dialysis catheter. Classic bicarbonate dialysis treatments lasted from 4 to 4.5 h and took place three to 5 times a week using high-flux dialyzers. The dialysis fluid flow was 500 mL/min, and the blood flow was 250–350 mL/min. The dialysis adequacy index (*Kt*/*V*) was 1.41 ± 0.23. Groups HD and C were similar demographically and anthropometrically. The study group and the control group were comparable with respect to age, sex, body weight, and BMI (Table 1).

### 3.1. Comparison of the Level of Anxiety in Patients on Dialysis (HD) and Controls with Normal Kidney Function (C)

The level of state anxiety in HD patients (X1) was significantly higher than in individuals in group C (35.1 ± 8.0 vs. 30.3 ± 7.0; *p* = 0.01). Trait anxiety (X2) level was higher in HD patients, but the difference was non-significant (41.8 ± 9.1 vs. 39.9 ± 9.4; *p* = 0.53) (Table 1).

### 3.2. Comparison of the Level of Anxiety in HD Patients with MS (HD-MS) and without MS (HD-nMS)

Differences between state anxiety and trait anxiety levels between HD patients with metabolic syndrome and without MS patients were statistically non-significant (respectively: 35.8 ± 7.9 vs. 34.5 ± 8.2, *p* = 0.43 for X1 and 41.7 ± 7.5 vs. 41.9 ± 10.7, *p* = 0.96 for X2) (Table 2).

### 3.3. Comparison of the Level of Anxiety in Controls with Normal Kidney Functions with Metabolic Syndrome (C-MS) and Controls with Normal Kidney Function without Metabolic Syndrome (C-nMS)

Differences in experiencing anxiety in terms of state anxiety severity between C-MS and C-nMS individuals with normal kidney function were statistically non-significant (respectively: 29.1 ± 6.6 vs. 31.2 ± 7.3, *p* = 0.37 for X1 and 39.7 ± 9.4 vs. 40.1 ± 9.7, *p* = 0.83 for X2) (Table 2).

### 3.4. Comparison of the Level of Anxiety in HD Patients and Individuals of the Control Group (C), Taking into Account the Presence or Absence of Metabolic Syndrome

The state anxiety level (X1) in HD-MS patients was considerably higher than in C-MS patients (35.8 ± 7.9 vs. 29.1 ± 6.6; *p* = 0.03). The difference was not apparent in patients without metabolic syndrome: the intensity of state anxiety in HD-nMS patients did not differ significantly from the control group (C-nMS) (34.5 ± 8.2 vs. 31.2 ± 7.3; *p* = 0.26).

In HD-MS patients, the intensity of trait anxiety (X2) was higher than in patients with normal renal function (C-MS) (41.7 ± 7.5 vs. 39.7 ± 9.4; *p* = 0.66), but it was not a statistically significant difference. The difference in trait anxiety intensity (X2) between the HD-nMS and C-nMS subgroups (41.9 ± 10.7 vs. 40.1 ± 9.7; *p* = 0.83) was also non-significant (Table 2).

### 3.5. The Influence of Physical Activity Planned and Adapted to the Patient’s Abilities on Tested Parameters

After four weeks of regular exercise in an inpatient ward, the level of state anxiety (X1) and trait anxiety (X2) lowered considerably in all HD patients (respectively: 35.1 ± 8.0 vs. 29.2 ± 5.0, *p* = 0.001 for X1 and 41.8 ± 9.1 vs. 38.1 ± 5.9, *p* = 0.008 for X2). A similar change was also observed in all subjects with normal renal function (respectively: 30.3 ± 7.0 vs. 27.8 ± 6.7, *p* = 0.05 for X1 and 39.9 ± 9.4 vs. 36.8 ± 7.2, *p* = 0.003 for X2).

After the rehabilitation program, there was no significant difference in the intensity of state anxiety observed in HD patients compared to patients with normal renal function, as observed before the program (29.2 ± 5.0 vs. 27.8 ± 6.7; *p* = 0.21) (Table 3). More data are provided in Supplementary Tables S1 and S3.

### 3.6. The Influence of Physical Activity Planned and Adapted to the Patient’s Abilities on Tested Parameters in Patients with Metabolic Syndrome

The rehabilitation program significantly reduced the intensity of anxiety state (X1) in HD-MS patients (35.8 ± 7.9 vs. 29.2 ± 5.1; *p* = 0.01). A similar change occurred in the C-MS group (29.1 ± 6.6 vs. 24.8 ± 3.5; *p* = 0.02). After the rehabilitation program, the difference in the severity of state anxiety (X1) between HD-MS and C-MS was still significant (29.2 ± 5.1 vs. 24.8 ± 3.5; *p* = 0.04).

After the rehabilitation program, a statistically significant reduction in trait anxiety level (X2) was observed in subjects with normal C-MS renal function (39.7 ± 9.4 vs. 35.6 ± 6.5; *p* = 0.04). In the HD-MS subgroup, the intensity of trait anxiety (X2) also decreased. However, this change was minimal (41.7 ± 7.5 vs. 39.3 ± 5.8; *p* = 0.10). After the rehabilitation program, as before the program, the difference in the intensity of trait anxiety (X2) between HD-MS and C-MS was non-significant (Table 4). More data are provided in Supplementary Tables S2 and S4.

### 3.7. The Influence of Physical Activity Planned and Adapted to the Patient’s Abilities on the Tested Parameters in Patients without Metabolic Syndrome

In subjects without the metabolic syndrome: HD-nMS and C-nMS, the rehabilitation program resulted in a statistically insignificant reduction in the intensity of anxiety state (X1) (respectively: 34.5 ± 8.2 vs. 29.1 ± 5.1, *p* = 0.09 for HD-nMS and 31.2 ± 7.3 vs. 30.1 ± 7.7, *p* = 0.54 for C-nMS). After the rehabilitation program, the intensity of state anxiety (X1) in HD-nMS patients was lower than u C-nMS, but the difference was not significant (29.1 ± 5.1 vs. 30.1 ± 7.7; *p* = 0.95).

The rehabilitation program helped to significantly reduce the intensity of trait anxiety (X2) in HD-nMS patients (41.9 ± 10.7 vs. 36.9 ± 5.9; *p* = 0.04). In patients with normal renal function without metabolic syndrome (C-nMS), the intensity of anxiety trait (X2) decreased not significantly (40.1 ± 9.7 vs. 37.7 ± 7.8; *p* = 0.08). The difference in the intensity of trait anxiety (X2) between HD-nMS and C-nMS was still non-significant (Table 5). More data are provided in Supplementary Tables S2 and S4.

## 4. Discussion

The main goal of our study was to determine whether the changes in anxiety intensity in HD patients and those with normal kidney function following the four-week rehabilitation program differed depending on the presence of MS. None of the previous studies on the relationship between physical activity and anxiety in HD patients considered the possible differences associated with MS.

An increasing number of authors emphasize the relationship between psychological conditions, especially anxiety and depression, with the development of somatic diseases and the influence of somatic disorders, especially chronic ones, on the occurrence of mental illnesses. Vasilopoulou et al. showed that a significant percentage of HD patients experienced a high level of anxiety or suffered from depression (47.8%; 38.2%, respectively) [54]. Epidemiological studies have shown that the life expectancy of patients with severe mental disorders is reduced by 7 to 24 years. Anxiety is one of the strongest predictors of depression. Many studies have confirmed the relationship between anxiety and mortality [55]. Therefore, it is beyond dispute that the search for methods of reducing distress in HD patients is one of the essential directions of care for this group of patients.

There have been many attempts to determine the relationship between anxiety and metabolic syndrome (MS) in subjects with normal kidney function [56]. Most of the authors suggested the need for further prospective studies to determine a two-way relationship between these clinical conditions [57]. Intervention studies in subjects without renal failure distinctly indicate that regular exercise can reduce anxiety symptoms and possibly depression [46,47,48,58].

The present study shows that the level of anxiety-state (X1) was higher in HD patients with metabolic syndrome (HD-MS) than in individuals with normal renal function (C-MS). Interestingly, in the absence of metabolic syndrome in HD patients, state anxiety (X1) did not differ significantly from state anxiety (X1) in subjects with normal kidney function. That indicates a significant influence of MS in the development of state anxiety in the studied patients.

The results of studies conducted so far, which did not consider the presence of the metabolic syndrome, indicate similarly to our observations that the intensity of anxiety in patients with uremia is significantly higher than in patients with normal renal function [59]. However, this study took into account the division into state anxiety and trait anxiety and has shown that the intensity of trait anxiety (X2) did not differ significantly in the entire group of HD patients compared to those with normal renal function.

The essential element of the presented work was an intervention consisting of a four-week, well-controlled physical effort adjusted to the patient’s abilities. The psychological effects of exercise in overweight or obese people are known [60]. Exercise alleviates symptoms of depression and anxiety and improves the quality of life [61,62,63].

The state anxiety (X1) decreased after four weeks of exercise, both in HD-MS patients and those with normal renal function (C-MS), but this change was significant only in patients with MS. The different response to physical exercise in HD patients in the sense of state anxiety is interesting (X1). Patients with metabolic syndrome benefited, in this case, more than patients without metabolic syndrome. State anxiety is a momentary and transient emotional response. The difference may be related to the rapid improvement in overall physical performance and weight loss with daily exercise, which is more noticeable in patients with MS. It may also be possible to improve your body image and increase your self-esteem after four weeks of regular exercise.

Trait anxiety which is understood as an individual and relatively permanent disposition to react with anxiety and perceive a given situation as threatening was also assessed. Although the differences between the initial and final severity of trait anxiety were significant in all HD patients and with normal renal function, differences in the severity of trait anxiety were observed depending on the presence of MS. In the absence of the metabolic syndrome, the change in the intensity of trait anxiety was significant in hemodialysis patients (HD-nMS) and subjects with normal renal function trait anxiety decreased only in patients with diagnosed metabolic syndrome (C-MS). Based on the above observations, it can be cautiously assumed that in terms of the impact of rehabilitation on the perception of trait anxiety (X2), there are differences due to the presence or absence of metabolic syndrome between HD patients and those with normal kidney function. This could suggest that non-MS HD patients benefit more in terms of reducing the severity of trait anxiety (X2) compared to patients with metabolic syndrome. This may be clinically relevant because, as shown in other studies [64], trait anxiety (X2) is most strongly associated with depressive symptoms.

In subjects with normal renal function, the subgroup with features of the metabolic syndrome showed more prominent benefits after the rehabilitation program in terms of state anxiety (X1) and trait anxiety (X2). The impact of exercise on anxiety in HD patients has been the subject of few studies so far. Our observations on the influence of physical activity on the feeling of distress confirmed the earlier reports of other authors, who suggested that regular physical exercise improves mood primarily when it is lowered and reduces the intensity of anxiety [46,47,48].

The general mechanisms of the influence of physical activity on reducing depression and anxiety disorders in various populations have been the subject of many studies. The presented hypotheses are primarily based on two theories: psychological and biological. The explanation of beneficial changes after exercise refers to two psychological trends: self-efficacy and the so-called the theory of distractors [65,66,67]. The change in self-esteem helps to regain conviction about the possibility of controlling one’s behavior and achieve personal goals [68]. The aim of distractors is to take your attention away from fears, worries, and other negative psychological stimuli. Among the biological hypotheses, the β-endorphin theory stands out. After exercise, an increase in the serum concentration of β-endorphin metabolites released by the pituitary gland was found [69,70]. Another attempt to explain the improvement of mood after exercise may be the thermogenic theory. Body temperature increases during physical exertion, which may reduce muscle and mental tension [71]. Studies by other authors have also shown the effect of exercise on the hypothalamic-pituitary-adrenal (HPA) system. The impact depends on the training load. Intense exercise causes a significant stimulation of the HPA axis. Long-term exercise with lighter loads reduces the reactivity of HPA [72]. The effect of physical exercise on improving mood may also be the result of the direct effect of exercise on increasing serotonin levels [73]. Moreover, physical exercise reduces fatigue and pain in the musculoskeletal system, increases muscle strength, and at the same time improves physical and mental performance [30,73,74] and has a beneficial effect on the cardiovascular system [29].

Regular physical activity leads to a decreased concentration of C-reactive protein and the concentration of pro-inflammatory cytokines such as IL-6 and TNF-α. However, data on a significant reduction of pro-inflammatory cytokines after physical activity programs in HD patients are inconclusive [49,75]. Reducing the concentration of pro-inflammatory cytokines may improve the functioning of the neurotransmitter system responsible for regulating mood—mainly serotonin, noradrenaline, and dopamine. That is consistent with the inflammatory theory of depression. However, it is not possible to state without any doubt that improved mood in subjects participating in the four-week rehabilitation program was the result of solely increased physical activity. The conditions during the stay in the rehabilitation department differed from those in everyday life. That could have contributed to improving the mental state. It is safe to assume that the number of stressors in everyday life has decreased. Increased social contacts could also have had a positive effect on the patient’s mood. The patients’ attitude towards improving their well-being was also essential. Psychology refers to it as a self-fulfilling prophecy. Many positive changes in tension and anxiety were also observed by other researchers after various forms of physical activity [26,27,28]. On the other hand, the rehabilitation program involved a stay in a rehabilitation ward, which could exacerbate the symptoms of anxiety and depression.

Other authors observed a beneficial effect of physical activity in reducing anxiety in patients with various chronic clinical conditions [76,77]. However, there is still little data confirming these observations in HD patients [78]. Dziubek et al. [79] assessed the impact of a six-month regular exercise program on symptoms of depression and anxiety during the HD procedure. State anxiety lowered after endurance exercise, and there was a reverse response after resistance exercise. Hemodialysis is one of the sources of distress. It may have been more difficult for the authors of this study to achieve favorable changes in the perception of resistance exercise during HD. Unlike in our research, the intensity of trait anxiety has not changed significantly. Another difference between our research and that of other authors was that our patients performed the exercises outside HD procedures as part of a stationary rehabilitation program. While some studies indicate a relationship between metabolic syndrome and depression [7,8], there is much less data on the relationship between MS and anxiety disorders and anxiety [9,10,77]. However, many researchers unanimously emphasize that distress should be recognized and treated as soon as possible [54].

Based on the conducted research, it is difficult to clearly explain the differentiation of the psychological effects of the four-week rehabilitation program based on the presence or absence of metabolic syndrome. The duration of the positive change in anxiety reduction was also not established. That could guide future research. It is likely that there are many direct and indirect mechanisms explaining the effect of MS on differences in response to exercise.

The presented study had several limitations. It was conducted in a relatively small group of subjects, and the observation period was relatively short. Moreover, the study included patients from one center, which makes generalizations difficult. However, single-center studies have allowed us to eliminate confounding factors associated with different standards and procedures.

The strength of our study was the way the exercises were managed. They occurred daily according to an individually selected scheme, during a four-week stay in stationary conditions, which guaranteed constant supervision of a multidisciplinary team and obtaining a very high level of compliance in the range of 90% to 100%. Thus far, this study design has not been employed in HD patients in any available reports. Another strong point of the study was having full access to information on the treatment of HD patients and maintaining the same methodology of studies conducted directly by the same researchers. The added value of the study was also the preparation and motivation of patients for regular physical activity after the end of the rehabilitation program.

These observations are of practical importance. They can play a role in creating specific recommendations for exercise in HD patients with MS. They also draw attention to the need for a more accurate diagnosis of patients with anxiety disorders and a more precise selection of measures to reduce the intensity of anxiety, taking into account the presence of metabolic syndrome. Clinical evaluation of anxiety disorders should be performed repeatedly: at the start of HD, as part of the annual mental health assessment when coexisting depression is suspected after critical life stressors occur, whenever there is a marked change in patients’ health, when treatment is not adhered to, or when there is unexplained and bothersome behavior by the patient in the dialysis station.

The actions reducing the perception of anxiety are all the more important as, according to other authors, depressive disorders and the accompanying distress that arises from complications of chronic kidney disease increase mortality, worsen the quality of life of patients, and contribute to non-adherence to treatment recommendations [80,81]. Ongoing anxiety assessment can help to stratify risk and identify patients at risk of death and cardiovascular morbidity [25]. The role of metabolic syndrome in response to exercise in HD patients should guide future research.

To summarize: after a four-week rehabilitation program, the severity of anxiety decreased in both HD patients and individuals with normal kidney function. In HD patients, the intensity of state anxiety was higher than in those with normal kidney function. The level of trait anxiety in HD patients did not differ from that in individuals with normal renal function. Unlike in the group with normal kidney function, the presence of MS was associated with higher levels of state anxiety in HD patients as compared to controls with normal renal function. The response to the four-week supervised rehabilitation program was different in HD patients as compared to controls with normal renal function. Beneficial reduction of trait anxiety (X2) was observed in HD-nMS patients. That was not the case for controls with normal renal function, among whom a considerable reduction of trait anxiety severity (X2) was associated with the presence of MS. It is important to note that the state anxiety (X1) significantly decreased both in HD patients and in individuals with normal kidney function only in the presence of MS.

This study enhances the insufficient literature on the effect of physical activity on reducing level of anxiety in HD patients, including metabolic syndrome. The research has scientific implications, especially in the context of reverse epidemiology in patients with end-stage renal disease. This study indicates the need to consider the presence or absence of MS in predicting the effect of physical activity on anxiety reduction in HD patients. The way the dialysis staff and HD patients understand the importance of physical activity in reducing the level of anxiety may fortify the effort to promote regular nephrological rehabilitation programs and physical activity during dialysis and between dialysis periods.

Our study did not analyze how long the reduced anxiety effect lasted after the rehabilitation program. Future research should identify the duration of the beneficial changes and other potential factors that influence the relationship between the effectiveness of physical activity in reducing anxiety.

## 5. Conclusions

1. Four-week physical activity planned and adapted to the patient’s abilities reduces the intensity of anxiety in HD patients and controls with normal renal function.
2. HD patients with MS benefit more with regard to reducing perceived state anxiety and HD patients without MS in terms of reducing trait anxiety.
3. In patients with normal kidney function without metabolic syndrome, the rehabilitation program did not significantly reduce state anxiety and trait anxiety, unlike patients with normal kidney function with metabolic syndrome in whom anxiety state and trait anxiety decreased.

## Figures and Tables

**Table 1:** Baseline characteristics of patients participating in the study (*n* = 58).

Variables	Study Group (HD)	Control Group (C)	*p*-Value
Age (years)	56.9 ± 13.3 (range = 25–75)	61.5 ± 8.3 (range = 37–76)	0.12
Sex, *n* (%) female/male	16 (57)/12 (43)	15 (50)/15 (50)	0.61
Dialysis vintage (months)	50.6 ± 73.4 (range = 5–272)	-	
Frequency of dialysis, *n* (%)		-	
3 times per week	24 (85.7)		
4 times per week	3 (10.7)		
5 times per week	1 (3.6)		
Dialysis adequacy (*Kt*/*V*)	1.41 ± 0.23	-	
BMI (kg/m^2^)	27.1 ± 6.08 (range = 16.4–42.1)	29.2 ± 5.3 (range = 21.3–41.0)	0.16
Weight (kg)	74.1 ± 20.1 (range = 43–126)	79.9 ± 17.7 (range = 53–117)	0.25
Vascular access, n (%)		-	
arteriovenous fistulas	24 (85.6)		
arteriovenous grafts	2 (7.2)		
central venous catheters	2 (7.2)		
Cause of end-stage kidney disease, *n* (%)		-	
glomerulonephritis	7 (25.1)		
diabetic kidney disease	6 (21.4)		
hypertension nephropathy	6 (21.4)		
other	9 (32.1)		
CCI (point)	4.5 ± 3.6 (range = 2–13)	1.03 ± 0.61 (range = 0–2)	0.001
Metabolic syndrome, *n* (%)	14 (50)	13 (43.3)	0.79
Diabetes, *n* (%)	7 (25)	4(14.3)	0.32
Waist circumference (cm)	96.3 ± 17.0 (range = 72–126)	96.7 ± 13.4 (range = 77–129)	0.92
Estimated GFR (mL/min/1.73 m^2^)	9.3 ± 3.6	97.6 ± 28.1	<0.001
Hemoglobin (g/dL)	11.16 ± 1.34	13.85 ± 0.81	<0.001
Uric acid (mg/dL)	5.24 ± 1.44	5.96 ± 1.58	0.08
Creatinine (mg/dL)	6.750 ± 2.435	0.843 ± 0.156	<0.001
Phosphorus (mg/dL)	5.234 ± 1.264	1.277 ± 0.209	<0.001
Sodium (mmol/dL)	139.6 ± 2.9	139.4 ± 2.0	0.70
Potassium (mmol/dL)	5.230 ± 0.522	4.743 ± 0.431	<0.001
PTH (pg/mL)	210.1 ± 187.8	55.41 ± 25.39	<0.001
X1	35.1 ± 8.0	30.3 ± 7.0	0.01
X2	41.8 ± 9.1	39.9 ± 9.4	0.53

Note: Results are the mean ± standard deviation (SD) or numbers (and percentages); significant at *p*-value <0.05. Abbreviations: BMI, body mass index; CCI, Charlson Comorbidity Index; GFR, glomerular filtration rate; PTH, parathyroid hormone; X1, state anxiety; X2, trait anxiety.

**Table 2:** Baseline comparison of the level of anxiety in the study (HD) and control group (C) with metabolic syndrome (MS) and without metabolic syndrome (nMS).

	Study Group (HD)		Control Group (C)		*p*-Value HD vs. C	
	HD-MS *n* = 14	HD-nMS *n* = 14	C-MS *n* = 13	C-nMS *n* = 17	HD-MS vs. C-MS	HD-nMS vs. C-nMS
X1 ($\bar{x}$ ± SD)	35.8 ± 7.9	34.5 ± 8.2	29.1 ± 6.6	31.2 ± 7.3	0.03	0.26
	*p* = 0.43		*p* = 0.37			
X2 ($\bar{x}$ ± SD)	41.7 ± 7.5	41.9 ± 10.7	39.7 ± 9,4	40.1 ± 9.7	0.66	0.84
	*p* = 0.96		*p* = 0.83			

Note: Results are the mean ± SD; significant at *p*-value < 0.05. Abbreviations: X1, state anxiety; X2, trait anxiety; HD, hemodialysis patients; C, patients with normal renal function; HD-MS, hemodialysis patients with metabolic syndrome; HD-nMS, hemodialysis patients without metabolic syndrome; C-MS, patients with normal renal function with metabolic syndrome; C-nMS, patients with normal renal function without metabolic syndrome.

**Table 3:** Effect of rehabilitation program on the studied parameters in study (HD) and control group (C).

	Study Group (HD)		Control Group (C)		*p*-ValueHD vs. C	
	Before	After	Before	After	Before	After
X1 ($\bar{x}$ ± SD)	35.1 ± 8.0	29.2 ± 5.0	30.3 ± 7.0	27.8 ± 6.7	0.01	0.21
	*p* = 0.001		*p* = 0.05			
X2 ($\bar{x}$ ± SD)	41.8 ± 9.1	38.1 ± 5.9	39.9 ± 9.4	36.8 ± 7.2	0.53	0.65
	*p* = 0.008		*p* = 0.003			

Note: Results are the mean ± SD; significant at *p*-value < 0.05. Abbreviations: X1, state anxiety; X2, trait anxiety; HD, hemodialysis patients; C, patients with normal renal function.

**Table 4:** Effect of rehabilitation program on the studied parameters in the study (HD) and control group (C) with metabolic syndrome (MS).

	Study Group (HD-MS)		Control Group (C-MS)		*p*-Value HD-MS vs. C-MS	
	Before	After	Before	After	Before	After
X1 ($\bar{x}$ ± SD)	35.8 ± 7.9	29.2 ± 5.1	29.1 ± 6.6	24.8 ± 3.5	0.03	0.04
	*p* = 0.01		*p* = 0.02			
X2 ($\bar{x}$ ± SD)	41.7 ± 7.5	39.3 ± 5.8	39.7 ± 9.4	35.6 ± 6.5	0.66	0.21
	*p* = 0.10		*p* = 0.04			

Note: Results are the mean ± SD; significant at *p*-value < 0.05. Abbreviations: X1, state anxiety; X2, trait anxiety; HD-MS, hemodialysis patients with metabolic syndrome; C-MS, patients with normal renal function with metabolic syndrome.

**Table 5:** Effect of rehabilitation program on the studied parameters in the study (HD) and control group (C) without metabolic syndrome (nMS).

	Study Group (HD-nMS)		Control Group (C-nMS)		*p*-Value HD-nMS vs. C-nMS	
	Before	After	Before	After	Before	After
X1 ($\bar{x}$ ± SD)	34.5 ± 8.2	29.1 ± 5.1	31.2 ± 7.3	30.1 ± 7.7	0.26	0.95
	*p* = 0.09		*p* = 0.54			
X2 ($\bar{x}$ ± SD)	41.9 ± 10.7	36.9 ± 5.9	40.1 ± 9.7	37.7 ± 7.8	0.84	0.56
	*p* = 0.04		*p* = 0.08			

Note: Results are the mean ± SD; significant at *p*-value < 0.05. Abbreviations: X1, state anxiety; X2, trait anxiety; HD-nMS, hemodialysis patients without metabolic syndrome; C-nMS, patients with normal renal function without metabolic syndrome.

## Data Availability

The results of scientific studies are anonymized and will be stored as such in an electronic form in a structured Statistica format on the premises of the facility conducting the study. The data will be stored for a period of 15 years. If requested, it can be made available in an anonymized form to the publisher within the scope of the presented work. Personal data related to this research are kept on the premises of the medical facility where the studies have been conducted. The data are subject to provisions on data protection in accordance with the law (Patients’ Rights and Patients’ Rights Ombudsman Article 26 Section 4).

## Supplementary Materials

The following are available online at https://www.mdpi.com/article/10.3390/ijerph182211952/s1. Table S1: Comparison of items of the STAI questionnaire before and after the rehabilitation program in the study and control group. Table S2: Comparison of items of the STAI questionnaire before and after the rehabilitation program in the study and control group with metabolic syndrome and without metabolic syndrome. Table S3: Comparison of mean values of items of the STAI questionnaire before and after the rehabilitation program in the study and control group. Table S4: Comparison of mean values of items of the STAI questionnaire before and after the rehabilitation program in the study and control group with metabolic syndrome and without metabolic syndrome.
