# Supplementary material for: Metabolic Syndrome and Psychological Effects of Exercise in Hemodialysis Patients
Source: Int J Environ Res Public Health. 2021 Nov 14;18(22):11952. doi: 10.3390/ijerph182211952 (PMC8622865; doi:10.3390/ijerph182211952)
Supplement: Supplementary file 1 [file ijerph-18-11952-s001.zip › ijerph-1427376-supplementary.pdf]

International Journal of
Environmental Research and Public Health

Supplementary Material

Table S1. Comparison of state (STAI-S) and trait anxiety (STAI-T) items of the STAI questionnaire before and after the rehabilitation program in the study (HD) and control group (C).

Statements			Study group (HD)		Control group (C)	
			Before	After	Before	After
			STAI-S			
1.	I feel calm	1	0	0	0	0
		2	2 (7.1)	1 (3.6)	2 (6.7)	0
		3	13 (46.4)	9 (32.1)	6 (20)	10 (33.3)
		4	13 (46.4)	18 (64.3)	22 (73.3)	20 (66.7)
2.	I feel secure	1	0	0	0	0
		2	0	0	0	0
		3	13 (46.4)	11 (39.3)	6 (20)	6 (20)
		4	15 (53.6)	17 (60.7)	24 (80)	24 (80)
3.	I am tense	1	15 (53.6)	17 (60.7)	19 (63.3)	21 (70)
		2	9 (32.1)	10 (35.7)	8 (26.7)	9 (30)
		3	3 (10.7)	1 (3.6)	3 (10)	0
		4	1 (3.6)	0	0	0
4.	I am regretful	1	13 (46.4)	23 (82.1)	22 (73.3)	26 (86.7)
		2	9 (32.1)	4 (14.3)	7 (23.3)	4 (13.3)
		3	3 (10.7)	1 (3.6)	1 (3.3)	0
		4	3 (10.7)	0	0	0
5.	I feel ease	1	0	2 (7.1)	0	0
		2	0	0	1 (3.3)	1 (3.3)
		3	15 (53.6)	9 (32.1)	12 (40)	4 (13.3)
		4	13 (46.4)	17 (60.7)	17 (56.7)	25 (83.3)
6.	I feel upset	1	11 (39.3)	23 (82.1)	23 (76.7)	25 (83.3)
		2	9 (32.1)	5 (17.9)	4 (13.3)	3 (10)
		3	5 (17.9)	0	3 (10)	2 (6.7)
		4	3 (10.7)	0	0	0
7.	I am presently worrying over possible misfortunes	1	12 (42.9)	15 (53.6)	19 (63.3)	20 (66.7)
		2	8 (28.6)	9 (32.1)	5 (16.7)	7 (23.3)
		3	4 (14.3)	2 (7.1)	4 (13.3)	3 (10)
		4	4 (14.3)	2 (7.1)	2 (6.7)	0
8.	I feel rested	1	4 (14.3)	0	1 (3.3)	0
		2	4 (14.3)	3 (10.7)	7 (23.3)	5 (16.7)
		3	12 (42.9)	6 (21.4)	12 (40)	11 (36.7)
		4	8 (28.6)	19 (67.9)	10 (33.3)	14 (46.7)
9.	I feel anxious	1	14 (50)	19 (67.9)	22 (73.3)	24 (80)
		2	10 (35.7)	8 (28.6)	6 (20)	2 (6.7)
		3	3 (10.7)	1 (3.6)	2 (6.7)	4 (13.3)
		4	1 (3.6)	0	0	0
10.	I feel comfortable	1	1 (3.6)	0	0	0
		2	2 (7.1)	0	2 (6.7)	1 (3.3)
		3	16 (57.1)	15 (53.6)	19 (63.3)	13 (43.3)
		4	9 (32.1)	13 (46.4)	9 (30)	16 (53.3)

11.	I feel self-confident	1	1 (3.6)	0	1 (3.3)	0
		2	7 (25)	5 (17.9)	3 (10)	4 (13.3)
		3	18 (64.3)	15 (53.6)	17 (56.7)	14 (46.7)
		4	2 (7.1)	8 (28.6)	9 (30)	12 (40)
12.	I feel nervous	1	21 (75)	22 (78.6)	23 (76.7)	22 (73.3)
		2	3 (10.7)	6 (21.4)	7 (23.3)	7 (23.3)
		3	2 (7.1)	0	0	1 (3.3)
		4	2 (7.1)	0	0	0
13.	I feel jittery	1	24 (85.7)	26 (92.9)	26 (86.7)	28 (93.3)
		2	3 (10.7)	2 (7.1)	4 (13.3)	2 (6.7)
		3	1 (3.6)	0	0	0
		4	0	0	0	0
14.	I feel "high-strung"	1	22 (78.6)	25 (89.3)	26 (86.7)	29 (96.7)
		2	4 (14.3)	1 (3.6)	4 (13.3)	0
		3	0	2 (7.1)	0	1 (3.3)
		4	2 (7.1)	0	0	0
15.	I am relaxed	1	1 (3.6)	0	0	0
		2	2 (7.1)	0	4 (13.3)	3 (10)
		3	19 (67.9)	18 (64.3)	15 (50)	14 (46.7)
		4	6 (21.4)	10 (35.7)	11 (36.7)	13 (43.3)
16.	I feel content	1	0	0	0	0
		2	2 (7.1)	2 (7.1)	1 (3.3)	0
		3	18 (64.3)	12 (42.9)	17 (56.7)	16 (53.3)
		4	8 (28.6)	14 (50)	12 (40)	14 (46.7)
17.	I am worried	1	10 (35.7)	18 (64.3)	19 (63.3)	21 (70)
		2	11 (39.3)	10 (35.7)	8 (26.7)	6 (20)
		3	4 (14.3)	0	3 (10)	3 (10)
		4	3 (10.7)	0	0	0
18.	I feel over excited and rattled	1	20 (71.4)	19 (67.9)	23 (76.7)	21 (70)
		2	8 (28.6)	7 (25)	6 (20)	6 (20)
		3	0	2 (7.1)	1 (3.3)	2 (6.7)
		4	0	0	0	1 (3.3)
19.	I feel joyful	1	0	0	0	0
		2	6 (21.4)	4 (14.3)	6 (20)	4 (13.3)
		3	21 (75)	19 (67.9)	18 (60)	13 (43.3)
		4	1 (3.6)	5 (17.9)	6 (20)	13 (43.3)
20.	I feel pleasant	1	1 (3.6)	0	0	0
		2	2 (7.1)	2 (7.1)	3 (10)	0
		3	20 (71.4)	17 (60.7)	23 (76.7)	16 (53.3)
		4	5 (17.9)	9 (32.1)	4 (13.3)	14 (46.7)
STAI-T						
21.	I feel pleasant	1	0	0	0	0
		2	12 (42.9)	10 (35.7)	8 (26.7)	10 (33.3)
		3	13 (46.4)	13 (46.4)	19 (63.3)	14 (46.7)
		4	3 (10.7)	5 (17.9)	3 (10)	6 (20)
22.	I tire quickly	1	1 (3.6)	4 (14.3)	6 (20)	7 (23.3)
		2	10 (35.7)	10 (35.7)	10 (33.3)	16 (53.3)
		3	9 (32.1)	14 (50)	10 (33.3)	7 (23.3)
		4	8 (28.6)	0	4 (13.3)	0

23.	I feel like crying	1	13 (46.4)	9 (32.1)	13 (43.3)	16 (53.3)
		2	10 (35.7)	17 (60.7)	13 (43.3)	13 (43.3)
		3	4 (14.3)	2 (7.1)	3 (10)	1 (3.3)
		4	1 (3.6)	0	1 (3.3)	0
24.	I wish I could be as happy as others seem to be	1	4 (14.3)	5 (17.9)	11 (36.7)	12 (40)
		2	7 (25)	9 (32.1)	10 (33.3)	7 (23.3)
		3	0	5 (17.9)	4 (13.3)	6 (20)
		4	17 (60.7)	9 (32.1)	5 (16.7)	5 (16.7)
25.	I am losing opportunities because I cannot make decisions fast	1	10 (35.7)	8 (28.6)	15 (50)	14 (46.7)
		2	11 (39.3)	12 (42.9)	9 (30)	12 (40)
		3	6 (21.4)	8 (28.6)	5 (16.7)	2 (6.7)
		4	1 (3.6)	0	1 (3.3)	2 (6.7)
26.	I feel rested	1	4 (14.3)	0	0	0
		2	9 (32.1)	9 (32.1)	12 (40)	4 (13.3)
		3	10 (35.7)	15 (53.6)	12 (40)	17 (56.7)
		4	5 (17.9)	4 (14.3)	6 (20)	9 (30)
27.	I am calm, cool, and collected	1	1 (3.6)	0	2 (6.7)	1 (3.3)
		2	6 (21.4)	4 (14.3)	9 (30)	5 (16.7)
		3	6 (21.4)	12 (42.9)	8 (26.7)	11 (36.7)
		4	15 (53.6)	12 (42.9)	11 (36.7)	13 (43.3)
28.	I feel that difficulties are piling up so that I cannot overcome them	1	8 (28.6)	12 (42.9)	13 (43.3)	13 (43.3)
		2	15 (53.6)	15 (53.6)	12 (40)	16 (53.3)
		3	4 (14.3)	1 (3.6)	5 (16.7)	1 (3.3)
		4	1 (3.6)	0	0	0
29.	I worry too much over something that really doesn't matter	1	12 (42.9)	14 (50)	7 (23.3)	10 (33.3)
		2	6 (21.4)	8 (28.6)	10 (33.3)	13 (43.3)
		3	7 (25)	6 (21.4)	9 (30)	4 (13.3)
		4	3 (10.7)	0	4 (13.3)	3 (10)
30.	I am happy	1	1 (3.6)	0	0	0
		2	12 (42.9)	9 (32.1)	10 (33.3)	8 (26.7)
		3	9 (32.1)	8 (28.6)	12 (40)	13 (43.3)
		4	6 (21.4)	11 (39.3)	8 (26.7)	9 (30)
31.	I am inclined to take things hard	1	10 (35.7)	6 (21.4)	14 (46.7)	13 (43.3)
		2	8 (28.6)	16 (57.1)	5 (16.7)	7 (23.3)
		3	6 (21.4)	3 (10.7)	8 (26.7)	8 (26.7)
		4	4 (14.3)	3 (10.7)	3 (10)	2 (6.7)
32.	I lack self-confidence	1	12 (42.9)	10 (35.7)	11 (36.7)	14 (46.7)
		2	13 (46.4)	16 (57.1)	10 (33.3)	13 (43.3)
		3	3 (10.7)	0	7 (23.3)	1 (3.3)
		4	0	2 (7.1)	2 (6.7)	2 (6.7)
33.	I feel secure	1	1 (3.6)	0	1 (3.3)	1 (3.3)
		2	3 (10.7)	2 (7.1)	6 (20)	4 (13.3)
		3	9 (32.1)	8 (28.6)	9 (30)	11 (36.7)
		4	15 (53.6)	18 (64.3)	14 (46.7)	14 (46.7)
34.	I try to avoid facing a crisis or difficulty	1	4 (14.3)	3 (10.7)	11 (36.7)	12 (40)
		2	12 (42.9)	13 (46.4)	8 (26.7)	9 (30)
		3	6 (21.4)	7 (25)	7 (23.3)	3 (10)
		4	6 (21.4)	5 (17.9)	4 (13.3)	6 (20)

35.	I feel blue	1	6 (21.4)	8 (28.6)	9 (30)	9 (30)
		2	15 (53.6)	17 (60.7)	17 (56.7)	20 (66.7)
		3	6 (21.4)	3 (10.7)	4 (13.3)	1 (3.3)
		4	1 (3.6)	0	0	0
36.	I am content	1	1 (3.6)	0	0	0
		2	12 (42.9)	9 (32.1)	9 (30)	9 (30)
		3	9 (32.1)	9 (32.1)	13 (43.3)	12 (40)
		4	6 (21.4)	10 (35.7)	8 (26.7)	9 (30)
37.	Some unimportant thought runs through my mind and bothers me	1	14 (50)	12 (42.9)	9 (30)	12 (40)
		2	10 (35.7)	14 (50)	15 (50)	15 (50)
		3	3 (10.7)	2 (7.1)	5 (16.7)	3 (10)
		4	1 (3.6)	0	1 (3.3)	0
38.	I take disappointments so keenly that I can't put them out of my mind	1	12 (42.9)	14 (50)	14 (46.7)	14 (46.7)
		2	7 (25)	10 (35.7)	8 (26.7)	9 (30)
		3	4 (14.3)	3 (10.7)	6 (20)	4 (13.3)
		4	5 (17.9)	1 (3.6)	2 (6.7)	3 (10)
39.	I am a steady person	1	0	0	1 (3.3)	0
		2	1 (3.6)	0	2 (6.7)	1 (3.3)
		3	7 (25)	11 (39.3)	12 (40)	10 (33.3)
		4	20 (71.4)	17 (60.7)	15 (50)	19 (63.3)
40.	I become tense and upset when I think about my current concerns	1	6 (21.4)	13 (46.4)	6 (20)	12 (40)
		2	16 (57.1)	11 (39.3)	17 (56.7)	10 (33.3)
		3	5 (17.9)	4 (14.3)	4 (13.3)	7 (23.3)
		4	1 (3.6)	0	3 (10)	1 (3.3)

Note: Results are numbers (and percentages). The Polish State and Trait Anxiety Inventory is an adaptation of the American State and Trait Anxiety Inventory test developed by C.D. Spielberger, R.L. Gorsuch and R.E. Lushene. The authors of the Polish adaptation of the test are J. Strelau, T. Tysarczyk and K. Wrześniewski. After the standardization and normalization works, the final version of the test was prepared by M. Tysarczyk. Abbreviations: STAI-S, State-Trait Anxiety Inventory – State; STAI-T, State-Trait Anxiety Inventory – Trait; HD, hemodialysis patients; C, patients with normal renal function. STAI-S scale: 1, not at all; 2, somewhat; 3, moderately so; 4, very much so. STAI-T scale: 1, almost never; 2, sometimes; 3, often; 4, almost always.

Table S2. Comparison of state (STAI-S) and trait anxiety (STAI-T) items of the STAI questionnaire before and after the rehabilitation program in the study (HD) and control group (C) with metabolic syndrome (MS) and without metabolic syndrome (nMS).

Statements		Study group (HD)				Control group (C)			
		Metabolic syndrome (MS)		Non-metabolic syndrome (nMS)		Metabolic syndrome (MS)		Non-metabolic syndrome (nMS)	
		Before	After	Before	After	Before	After	Before	After
STAI-S									
1. I feel calm	1	0	0	0	0	0	0	0	0
	2	1 (7.1)	0	1 (7.1)	1 (7.1)	0	0	2 (11.8)	0
	3	8 (57.1)	6 (42.9)	5 (35.7)	3 (21.4)	3 (23.1)	2 (15.4)	3 (17.6)	8 (47.1)
	4	5 (35.7)	8 (57.1)	8 (57.1)	10 (71.4)	10 (76.9)	11 (84.6)	12 (70.6)	9 (52.9)
2. I feel secure	1	0	0	0	0	0	0	0	0
	2	0	0	0	0	0	0	0	0
	3	8 (57.1)	5 (35.7)	5 (35.7)	6 (42.9)	3 (23.1)	1 (7.7)	3 (17.6)	5 (29.4)
	4	6 (42.9)	9 (64.3)	9 (64.3)	8 (57.1)	10 (76.9)	12 (92.3)	14 (82.4)	12 (70.6)
3. I am tense	1	8 (57.1)	10 (71.4)	7 (50)	7 (50)	9 (69.2)	11 (84.6)	10 (58.8)	10 (58.8)
	2	5 (35.7)	4 (28.6)	4 (28.6)	6 (42.9)	3 (23.1)	2 (15.4)	5 (29.4)	7 (41.2)
	3	1 (7.1)	0	2 (14.3)	1 (7.1)	1 (7.7)	0	2 (11.8)	0
	4	0	0	1 (7.1)	0	0	0	0	0
4. I am regretful	1	5 (35.7)	11 (78.6)	8 (57.1)	12 (85.7)	10 (76.9)	12 (92.3)	12 (70.6)	14 (82.4)
	2	7 (50)	3 (21.4)	2 (14.3)	1 (7.1)	3 (23.1)	1 (7.7)	4 (23.5)	3 (17.6)
	3	1 (7.1)	0	2 (14.3)	1 (7.1)	0	0	1 (5.9)	0
	4	1 (7.1)	0	2 (14.3)	0	0	0	0	0
5. I feel ease	1	0	1 (7.1)	0	1 (7.1)	0	0	0	0
	2	0	0	0	0	0	0	1 (5.9)	1 (5.9)
	3	7 (50)	4 (28.6)	8 (57.1)	5 (35.7)	5 (38.5)	0	7 (41.2)	4 (23.5)
	4	7 (50)	9 (64.3)	6 (42.9)	8 (57.1)	8 (61.5)	13 (100)	9 (52.9)	12 (70.6)
6. I feel upset	1	5 (35.7)	12 (85.7)	6 (42.9)	11 (78.6)	11 (84.6)	12 (92.3)	12 (70.6)	13 (76.5)
	2	4 (28.6)	2 (14.3)	5 (35.7)	3 (21.4)	2 (15.4)	1 (7.7)	2 (11.8)	2 (11.8)
	3	3 (21.4)	0	2 (14.3)	0	0	0	3 (17.6)	2 (11.8)
	4	2 (14.3)	0	1 (7.1)	0	0	0	0	0
7. I am presently worrying over possible misfortunes	1	6 (42.9)	8 (57.1)	6 (42.9)	7 (50)	9 (69.2)	10 (76.9)	10 (58.8)	10 (58.8)
	2	3 (21.4)	4 (28.6)	5 (35.7)	5 (35.7)	1 (7.7)	3 (23.1)	4 (23.5)	4 (23.5)
	3	2 (14.3)	2 (14.3)	2 (14.3)	0	2 (15.4)	0	2 (11.8)	3 (17.6)
	4	3 (21.4)	0	1 (7.1)	2 (14.3)	1 (7.7)	0	1 (5.9)	0
8. I feel rested	1	2 (14.3)	0	2 (14.3)	0	1 (7.7)	0	0	0
	2	2 (14.3)	0	2 (14.3)	3 (21.4)	2 (15.4)	2 (15.4)	5 (29.4)	3 (17.6)
	3	7 (50)	5 (35.7)	5 (35.7)	1 (7.1)	6 (46.2)	3 (23.1)	6 (35.3)	8 (47.1)
	4	3 (21.4)	9 (64.3)	5 (35.7)	10 (71.4)	4 (30.8)	8 (61.5)	6 (35.3)	6 (35.3)
9. I feel anxious	1	8 (57.1)	9 (64.3)	6 (42.9)	10 (71.4)	10 (76.9)	12 (92.3)	12 (70.6)	12 (70.6)
	2	4 (28.6)	4 (28.6)	6 (42.9)	4 (28.6)	2 (15.4)	1 (7.7)	4 (23.5)	1 (5.9)
	3	1 (7.1)	1 (7.1)	2 (14.3)	0	1 (7.7)	0	1 (5.9)	4 (23.5)
	4	1 (7.1)	0	0	0	0	0	0	0
10. I feel comfortable	1	1 (7.1)	0	0	0	0	0	12 (70.6)	0
	2	1 (7.1)	0	1 (7.1)	0	1 (7.7)	0	4 (23.5)	1 (5.9)
	3	8 (57.1)	9 (64.3)	8 (57.1)	6 (42.9)	8 (61.5)	5 (38.5)	1 (5.9)	8 (47.1)
	4	4 (28.6)	5 (35.7)	5 (35.7)	8 (57.1)	4 (30.8)	8 (61.5)	0	8 (47.1)

11. I feel self-confident	1	1 (7.1)	0	0	0	0	0	0	0
	2	4 (28.6)	2 (14.3)	3 (21.4)	3 (21.4)	1 (7.7)	0	1 (5.9)	4 (23.5)
	3	9 (64.3)	10 (71.4)	9 (64.3)	5 (35.7)	9 (69.2)	6 (46.2)	11 (64.7)	8 (47.1)
	4	0	2 (14.3)	2 (14.3)	6 (42.9)	3 (23.1)	7 (53.8)	5 (29.4)	5 (29.4)
12. I feel nervous	1	10 (71.4)	11 (78.6)	11 (78.6)	11 (78.6)	11 (84.6)	13 (100)	1 (5.9)	9 (52.9)
	2	3 (21.4)	3 (21.4)	0	3 (21.4)	2 (15.4)	0	2 (11.8)	7 (41.2)
	3	1 (7.1)	0	1 (7.1)	0	0	0	8 (47.1)	1 (5.9)
	4	0	0	2 (14.3)	0	0	0	6 (35.3)	0
13. I feel jittery	1	12 (85.7)	12 (85.7)	12 (85.7)	14 (100)	11 (84.6)	13 (100)	12 (70.6)	15 (88.2)
	2	2 (14.3)	2 (14.3)	1 (7.1)	0	2 (15.4)	0	5 (29.4)	2 (11.8)
	3	0	0	1 (7.1)	0	0	0	0	0
	4	0	0	0	0	0	0	0	0
14. I feel "high-strung"	1	11 (78.6)	12 (85.7)	11 (78.6)	13 (92.9)	11 (84.6)	13 (100)	15 (88.2)	16 (94.1)
	2	1 (7.1)	1 (7.1)	3 (21.4)	0	2 (15.4)	0	2 (11.8)	0
	3	0	1 (7.1)	0	1 (7.1)	0	0	0	1 (5.9)
	4	2 (14.3)	0	0	0	0	0	0	0
15. I am relaxed	1	0	0	1 (7.1)	0	0	0	15 (88.2)	0
	2	0	0	2 (14.3)	0	2 (15.4)	1 (7.7)	2 (11.8)	2 (11.8)
	3	11 (78.6)	10 (71.4)	8 (57.1)	8 (57.1)	6 (46.2)	4 (30.8)	0	10 (58.8)
	4	3 (21.4)	4 (28.6)	3 (21.4)	6 (42.9)	5 (38.5)	8 (61.5)	0	5 (29.4)
16. I feel content	1	0	0	0	0	0	0	0	0
	2	1 (7.1)	0	1 (7.1)	2 (14.3)	0	0	2 (11.8)	0
	3	10 (71.4)	7 (50)	8 (57.1)	5 (35.7)	8 (61.5)	6 (46.2)	9 (52.9)	10 (58.8)
	4	3 (21.4)	7 (50)	5 (35.7)	7 (50)	5 (38.5)	7 (53.8)	6 (35.3)	7 (41.2)
17. I am worried	1	4 (28.6)	9 (64.3)	6 (42.9)	9 (64.3)	10 (76.9)	11 (84.6)	0	10 (58.8)
	2	7 (50)	5 (35.7)	4 (28.6)	5 (35.7)	1 (7.7)	2 (15.4)	1 (5.9)	4 (23.5)
	3	1 (7.1)	0	3 (21.4)	0	2 (15.4)	0	9 (52.9)	3 (17.6)
	4	2 (14.3)	0	1 (7.1)	0	0	0	7 (41.2)	0
18. I feel over excited and rattled	1	11 (78.6)	11 (78.6)	9 (64.3)	8 (57.1)	11 (84.6)	10 (76.9)	12 (70.6)	11 (64.7)
	2	3 (21.4)	3 (21.4)	5 (35.7)	4 (28.6)	2 (15.4)	1 (7.7)	4 (23.5)	5 (29.4)
	3	0	0	0	2 (14.3)	0	1 (7.7)	1 (5.9)	1 (5.9)
	4	0	0	0	0	0	1 (7.7)	0	0
19. I feel joyful	1	0	0	0	0	0	0	0	0
	2	3 (21.4)	2 (14.3)	3 (21.4)	2 (14.3)	1 (7.7)	1 (7.7)	5 (29.4)	3 (17.6)
	3	11 (78.6)	12 (85.7)	10 (71.4)	7 (50)	8 (61.5)	6 (46.2)	10 (58.8)	7 (41.2)
	4	0	0	1 (7.1)	5 (35.7)	4 (30.8)	6 (46.2)	2 (11.8)	7 (41.2)
20. I feel pleasant	1	0	0	1 (7.1)	0	0	0	0	0
	2	1 (7.1)	0	1 (7.1)	2 (14.3)	0	0	3 (17.6)	0
	3	12 (85.7)	11 (78.6)	8 (57.1)	6 (42.9)	10 (76.9)	6 (46.2)	13 (76.5)	10 (58.8)
	4	1 (7.1)	3 (21.4)	4 (28.6)	6 (42.9)	3 (23.1)	7 (53.8)	1 (5.9)	7 (41.2)
STAI-T									
21. I feel pleasant	1	0	0	0	0	0	0	0	0
	2	8 (57.1)	7 (50)	4 (28.6)	3 (21.4)	3 (23.1)	4 (30.8)	5 (29.4)	6 (35.3)
	3	5 (35.7)	7 (50)	8 (57.1)	6 (42.9)	9 (69.2)	6 (46.2)	10 (58.8)	8 (47.1)
	4	1 (7.1)	0	2 (14.3)	5 (35.7)	1 (7.7)	3 (23.1)	2 (11.8)	3 (17.6)
22. I tire quickly	1	0	1 (7.1)	1 (7.1)	3 (21.4)	2 (15.4)	3 (23.1)	4 (23.5)	4 (23.5)
	2	4 (28.6)	6 (42.9)	6 (42.9)	4 (28.6)	4 (30.8)	6 (46.2)	6 (35.3)	10 (58.8)
	3	7 (50)	7 (50)	2 (14.3)	7 (50)	5 (38.5)	4 (30.8)	5 (29.4)	3 (17.6)
	4	3 (21.4)	0	5 (35.7)	0	2 (15.4)	0	2 (11.8)	0

23.	I feel like crying	1	7 (50)	4 (28.6)	6 (42.9)	5 (35.7)	7 (53.8)	9 (69.2)	6 (35.3)	7 (41.2)
		2	4 (28.6)	8 (57.1)	6 (42.9)	9 (64.3)	4 (30.8)	4 (30.8)	9 (52.9)	9 (52.9)
		3	2 (14.3)	2 (14.3)	2 (14.3)	0	2 (15.4)	0	1 (5.9)	1 (5.9)
		4	1 (7.1)	0	0	0	0	0	1 (5.9)	0
24.	I wish I could be as happy as others seem to be	1	2 (14.3)	1 (7.1)	2 (14.3)	4 (28.6)	3 (23.1)	4 (30.8)	8 (47.1)	8 (47.1)
		2	4 (28.6)	4 (28.6)	3 (21.4)	5 (35.7)	5 (38.5)	4 (30.8)	5 (29.4)	3 (17.6)
		3	0	3 (21.4)	0	2 (14.3)	3 (23.1)	2 (15.4)	1 (5.9)	4 (23.5)
		4	8 (57.1)	6 (42.9)	9 (64.3)	3 (21.4)	2 (15.4)	3 (23.1)	3 (17.6)	2 (11.8)
25.	I am losing opportunities because I cannot make decisions fast	1	5 (35.7)	6 (42.9)	5 (35.7)	2 (14.3)	7 (53.8)	8 (61.5)	8 (47.1)	6 (35.3)
		2	7 (50)	5 (35.7)	4 (28.6)	7 (50)	5 (38.5)	3 (23.1)	4 (23.5)	9 (52.9)
		3	2 (14.3)	3 (21.4)	4 (28.6)	5 (35.7)	1 (7.7)	1 (7.7)	4 (23.5)	1 (5.9)
		4	0	0	1 (7.1)	0	0	1 (7.7)	1 (5.9)	1 (5.9)
26.	I feel rested	1	1 (7.1)	0	3 (21.4)	0	0	0	0	0
		2	7 (50)	5 (35.7)	2 (14.3)	4 (28.6)	7 (53.8)	1 (7.7)	5 (29.4)	3 (17.6)
		3	4 (28.6)	8 (57.1)	6 (42.9)	7 (50)	4 (30.8)	8 (61.5)	8 (47.1)	9 (52.9)
		4	2 (14.3)	1 (7.1)	3 (21.4)	3 (21.4)	2 (15.4)	4 (30.8)	4 (23.5)	5 (29.4)
27.	I am calm, cool, and collected	1	0	0	1 (7.1)	0	0	0	2 (11.8)	1 (5.9)
		2	5 (35.7)	2 (14.3)	1 (7.1)	2 (14.3)	4 (30.8)	1 (7.7)	5 (29.4)	4 (23.5)
		3	2 (14.3)	9 (64.3)	4 (28.6)	3 (21.4)	4 (30.8)	6 (46.2)	4 (23.5)	5 (29.4)
		4	7 (50)	3 (21.4)	8 (57.1)	9 (64.3)	5 (38.5)	6 (46.2)	6 (35.3)	7 (41.2)
28.	I feel that difficulties are piling up so that I cannot overcome them	1	5 (35.7)	7 (50)	3 (21.4)	5 (35.7)	5 (38.5)	3 (23.1)	8 (47.1)	10 (58.8)
		2	8 (57.1)	6 (42.9)	7 (50)	9 (64.3)	5 (38.5)	10 (76.9)	7 (41.2)	6 (35.3)
		3	1 (7.1)	1 (7.1)	3 (21.4)	0	3 (23.1)	0	2 (11.8)	1 (5.9)
		4	0	0	1 (7.1)	0	0	0	0	0
29.	I worry too much over something that really doesn't matter	1	6 (42.9)	8 (57.1)	6 (42.9)	6 (42.9)	3 (23.1)	5 (38.5)	4 (23.5)	5 (29.4)
		2	4 (28.6)	2 (14.3)	2 (14.3)	6 (42.9)	3 (23.1)	6 (46.2)	7 (41.2)	7 (41.2)
		3	4 (28.6)	4 (28.6)	3 (21.4)	2 (14.3)	5 (38.5)	2 (15.4)	4 (23.5)	2 (11.8)
		4	0	0	3 (21.4)	0	2 (15.4)	0	2 (11.8)	3 (17.6)
30.	I am happy	1	0	0	1 (7.1)	0	0	0	0	0
		2	8 (57.1)	6 (42.9)	4 (28.6)	3 (21.4)	4 (30.8)	2 (15.4)	6 (35.3)	6 (35.3)
		3	5 (35.7)	3 (21.4)	4 (28.6)	5 (35.7)	6 (46.2)	9 (69.2)	6 (35.3)	4 (23.5)
		4	1 (7.1)	5 (35.7)	5 (35.7)	6 (42.9)	3 (23.1)	2 (15.4)	5 (29.4)	7 (41.2)
31.	I am inclined to take things hard	1	6 (42.9)	3 (21.4)	4 (28.6)	3 (21.4)	6 (46.2)	6 (46.2)	8 (47.1)	7 (41.2)
		2	4 (28.6)	9 (64.3)	4 (28.6)	7 (50)	2 (15.4)	5 (38.5)	3 (17.6)	2 (11.8)
		3	4 (28.6)	0	2 (14.3)	3 (21.4)	4 (30.8)	1 (7.7)	4 (23.5)	7 (41.2)
		4	0	2 (14.3)	4 (28.6)	1 (7.1)	1 (7.7)	1 (7.7)	2 (11.8)	1 (5.9)
32.	I lack self-confidence	1	6 (42.9)	7 (50)	6 (42.9)	3 (21.4)	6 (46.2)	5 (38.5)	5 (29.4)	9 (52.9)
		2	7 (50)	7 (50)	6 (42.9)	9 (64.3)	5 (38.5)	6 (46.2)	5 (29.4)	7 (41.2)
		3	1 (7.1)	0	2 (14.3)	0	1 (7.7)	1 (7.7)	6 (35.3)	0
		4	0	0	0	2 (14.3)	1 (7.7)	1 (7.7)	1 (5.9)	1 (5.9)
33.	I feel secure	1	1 (7.1)	0	0	0	1 (7.7)	1 (7.7)	0	0
		2	1 (7.1)	2 (14.3)	2 (14.3)	0	2 (15.4)	1 (7.7)	4 (23.5)	3 (17.6)
		3	5 (35.7)	3 (21.4)	4 (28.6)	5 (35.7)	3 (23.1)	4 (30.8)	6 (35.3)	7 (41.2)
		4	7 (50)	9 (64.3)	8 (57.1)	9 (64.3)	7 (53.8)	7 (53.8)	7 (41.2)	7 (41.2)
34.	I try to avoid facing a crisis or difficulty	1	2 (14.3)	2 (14.3)	2 (14.3)	1 (7.1)	3 (23.1)	4 (30.8)	8 (47.1)	8 (47.1)
		2	7 (50)	5 (35.7)	5 (35.7)	8 (57.1)	5 (38.5)	7 (53.8)	3 (17.6)	2 (11.8)
		3	3 (21.4)	4 (28.6)	3 (21.4)	3 (21.4)	3 (23.1)	0	4 (23.5)	3 (17.6)
		4	2 (14.3)	3 (21.4)	4 (28.6)	2 (14.3)	2 (15.4)	2 (15.4)	2 (11.8)	4 (23.5)

35.	I feel blue	1	2 (14.3)	5 (35.7)	4 (28.6)	3 (21.4)	6 (46.2)	6 (46.2)	3 (17.6)	3 (17.6)
		2	9 (64.3)	7 (50)	6 (42.9)	10 (71.4)	5 (38.5)	7 (53.8)	12 (70.6)	13 (76.5)
		3	3 (21.4)	2 (14.3)	3 (21.4)	1 (7.1)	2 (15.4)	0	2 (11.8)	1 (5.9)
		4	0	0	1 (7.1)	0	0	0	0	0
36.	I am content	1	0	0	1 (7.1)	0	0	0	0	0
		2	9 (64.3)	5 (35.7)	3 (21.4)	4 (28.6)	4 (30.8)	3 (23.1)	5 (29.4)	6 (35.3)
		3	3 (21.4)	5 (35.7)	6 (42.9)	4 (28.6)	5 (38.5)	6 (46.2)	8 (47.1)	6 (35.3)
		4	2 (14.3)	4 (28.6)	4 (28.6)	6 (42.9)	4 (30.8)	4 (30.8)	4 (23.5)	5 (29.4)
37.	Some unimportant thought runs through my mind and bothers me	1	7 (50)	6 (42.9)	7 (50)	6 (42.9)	3 (23.1)	5 (38.5)	6 (35.3)	7 (41.2)
		2	5 (35.7)	7 (50)	5 (35.7)	7 (50)	8 (61.5)	6 (46.2)	7 (41.2)	9 (52.9)
		3	1 (7.1)	1 (7.1)	2 (14.3)	1 (7.1)	2 (15.4)	2 (15.4)	3 (17.6)	1 (5.9)
		4	1 (7.1)	0	0	0	0	0	1 (5.9)	0
38.	I take disappointments so keenly that I can't put them out of my mind	1	5 (35.7)	7 (50)	7 (50)	7 (50)	8 (61.5)	7 (53.8)	6 (35.3)	7 (41.2)
		2	3 (21.4)	6 (42.9)	4 (28.6)	4 (28.6)	2 (15.4)	4 (30.8)	6 (35.3)	5 (29.4)
		3	4 (28.6)	1 (7.1)	0	2 (14.3)	2 (15.4)	2 (15.4)	4 (23.5)	2 (11.8)
		4	2 (14.3)	0	3 (21.4)	1 (7.1)	1 (7.7)	0	1 (5.9)	3 (17.6)
39.	I am a steady person	1	0	0	0	0	0	0	1 (5.9)	0
		2	0	0	1 (7.1)	0	0	0	2 (11.8)	1 (5.9)
		3	5 (35.7)	7 (50)	2 (14.3)	4 (28.6)	7 (53.8)	6 (46.2)	5 (29.4)	4 (23.5)
		4	9 (64.3)	7 (50)	11 (78.6)	10 (71.4)	6 (46.2)	7 (53.8)	9 (52.9)	12 (70.6)
40.	I become tense and upset when I think about my current concerns	1	3 (21.4)	5 (35.7)	3 (21.4)	8 (57.1)	2 (15.4)	6 (46.2)	4 (23.5)	6 (35.3)
		2	10 (71.4)	5 (35.7)	6 (42.9)	6 (42.9)	8 (61.5)	5 (38.5)	9 (52.9)	5 (29.4)
		3	1 (7.1)	4 (28.6)	4 (28.6)	0	2 (15.4)	2 (15.4)	2 (11.8)	5 (29.4)
		4	0	0	1 (7.1)	0	1 (7.7)	0	2 (11.8)	1 (5.9)

Note: Results are numbers (and percentages). The Polish State and Trait Anxiety Inventory is an adaptation of the American State and Trait Anxiety Inventory test developed by C.D. Spielberger, R.L. Gorsuch and R.E. Lushene. The authors of the Polish adaptation of the test are J. Strelau, T. Tysarczyk and K. Wrześniewski. After the standardization and normalization works, the final version of the test was prepared by M. Tysarczyk. Abbreviations: STAI-S, State-Trait Anxiety Inventory – State; STAI-T, State-Trait Anxiety Inventory – Trait; HD, hemodialysis patients; C, patients with normal renal function. STAI-S scale: 1, not at all; 2, somewhat; 3, moderately so; 4, very much so. STAI-T scale: 1, almost never; 2, sometimes; 3, often; 4, almost always.

Table S3. Comparison of mean values of state (STAI-S) and trait anxiety (STAI-T) items of the STAI questionnaire before and after the rehabilitation program in the study (HD) and control group (C).

	Statements	Study group (HD)		Control group (C)	
		**Before**	**After**	**Before**	**After**
	STAI-S				
1.	I feel calm*	1.61	1.39	1.33	1.33
2.	I feel secure*	1.46	1.39	1.20	1.20
3.	I am tense	1.64	1.43	1.47	1.30
4.	I am regretful	1.86	1.21	1.30	1.13
5.	I feel ease*	1.54	1.54	1.47	1.20
6.	I feel upset	2.00	1.18	1.33	1.23
7.	I am presently worrying over possible misfortunes	2.00	1.68	1.63	1.43
8.	I feel rested*	2.14	1.43	1.97	1.70
9.	I feel anxious	1.68	1.36	1.33	1.33
10.	I feel comfortable*	1.82	1.54	1.77	1.50
11.	I feel self-confident*	2.25	1.89	1.87	1.73
12.	I feel nervous	1.46	1.21	1.23	1.30
13.	I feel jittery	1.18	1.07	1.13	1.07
14.	I feel "high-strung"	1.36	1.18	1.13	1.07
15.	I am relaxed*	1.93	1.64	1.77	1.67
16.	I feel content*	1.79	1.57	1.63	1.53
17.	I am worried	2.00	1.36	1.47	1.40
18.	I feel over excited and rattled	1.29	1.39	1.27	1.43
19.	I feel joyful*	2.18	1.96	2.00	1.70
20.	I feel pleasant*	1.96	1.75	1.97	1.53
	STAI-T				
21.	I feel pleasant*	2.32	2.18	2.17	2.13
22.	I tire quickly	2.86	2.36	2.40	2.00
23.	I feel like crying	1.75	1.75	1.73	1.50
24.	I wish I could be as happy as others seem to be	3.07	2.64	2.10	2.13
25.	I am losing opportunities because I cannot make decisions fast	1.93	2.00	1.73	1.73
26.	I feel rested*	2.43	2.18	2.20	1.83
27.	I am calm, cool, and collected*	1.75	1.71	2.07	1.80
28.	I feel that difficulties are piling up so that I cannot overcome them	1.93	1.61	1.73	1.60
29.	I worry too much over something that really doesn't matter	2.04	1.71	2.33	2.00
30.	I am happy*	2.29	1.93	2.07	1.97
31.	I am inclined to take things hard	2.14	2.11	2.00	1.97
32.	I lack self-confidence	1.68	1.79	2.00	1.70
33.	I feel secure*	1.64	1.43	1.80	1.73
34.	I try to avoid facing a crisis or difficulty	2.50	2.50	2.13	2.10
35.	I feel blue	2.07	1.82	1.83	1.73
36.	I am content*	2.29	1.96	2.03	2.00
37.	Some unimportant thought runs through my mind and bothers me	1.68	1.64	1.93	1.70
38.	I take disappointments so keenly that I can't put them out of my mind	2.07	1.68	1.87	1.87
39.	I am a steady person*	1.32	1.39	1.63	1.40
40.	I become tense and upset when I think about my current concerns	2.04	1.68	2.13	1.90

Note: Results are mean values. Reverse counting items are shown with "*". The Polish State and Trait Anxiety Inventory is an adaptation of the American State and Trait Anxiety Inventory test developed by C.D. Spielberger, R.L. Gorsuch and R.E. Lushene. The authors of the Polish adaptation of the test are J. Strelau, T. Tysarczyk and K. Wrześniewski. After the standardization and normalization works, the final version of the test was prepared by M. Tysarczyk. Abbreviations: STAI-S, State-Trait Anxiety Inventory – State; STAI-T, State-Trait Anxiety Inventory – Trait; HD, hemodialysis patients; C, patients with normal renal function.

Table S4. Comparison of mean values of state (STAI-S) and trait anxiety (STAI-T) items of the STAI questionnaire before and after the rehabilitation program in the study (HD) and control group (C) with metabolic syndrome (MS) and without metabolic syndrome (nMS).

	Statements	Study group (HD)				Control group (C)			
		Metabolic syndrome (MS)		Non-metabolic syndrome (nMS)		Metabolic syndrome (MS)		Non-metabolic syndrome (nMS)	
		Before	After	Before	After	Before	After	Before	After
	STAI-S								
1.	I feel calm*	1.71	1.43	1.50	1.36	1.23	1.15	1.41	1.47
2.	I feel secure*	1.57	1.36	1.36	1.43	1.23	1.08	1.18	1.29
3.	I am tense	1.50	1.29	1.79	1.57	1.38	1.15	1.53	1.41
4.	I am regretful	1.86	1.21	1.86	1.21	1.23	1.08	1.35	1.18
5.	I feel ease*	1.50	1.50	1.57	1.57	1.38	1.00	1.53	1.35
6.	I feel upset	2.14	1.14	1.86	1.21	1.15	1.08	1.47	1.35
7.	I am presently worrying over possible misfortunes	2.14	1.57	1.86	1.79	1.62	1.23	1.65	1.59
8.	I feel rested*	2.21	1.36	2.07	1.50	2.00	1.54	1.94	1.82
9.	I feel anxious	1.64	1.43	1.71	1.29	1.31	1.08	1.35	1.53
10.	I feel comfortable*	1.93	1.64	1.71	1.43	1.77	1.38	1.76	1.59
11.	I feel self-confident*	2.43	2.00	2.07	1.79	1.85	1.46	1.88	1.94
12.	I feel nervous	1.36	1.21	1.57	1.21	1.15	1.00	1.29	1.53
13.	I feel jittery	1.14	1.14	1.21	1.00	1.15	1.00	1.12	1.12
14.	I feel "high-strung"	1.50	1.21	1.21	1.14	1.15	1.00	1.12	1.12
15.	I am relaxed*	1.79	1.71	2.07	1.57	1.77	1.46	1.76	1.82
16.	I feel content*	1.86	1.50	1.71	1.64	1.62	1.46	1.65	1.59
17.	I am worried	2.07	1.36	1.93	1.36	1.38	1.15	1.53	1.59
18.	I feel over excited and rattled	1.21	1.21	1.36	1.57	1.15	1.46	1.35	1.41
19.	I feel joyful*	2.21	2.14	2.14	1.79	1.77	1.62	2.18	1.76
20.	I feel pleasant*	2.00	1.79	1.93	1.71	1.77	1.46	2.12	1.59
	STAI-T								
21.	I feel pleasant*	2.50	2.50	2.14	1.86	2.15	2.08	2.18	2.18
22.	I tire quickly	2.93	2.43	2.79	2.29	2.54	2.08	2.29	1.94
23.	I feel like crying	1.79	1.86	1.71	1.64	1.62	1.31	1.82	1.65
24.	I wish I could be as happy as others seem to be	3.00	3.00	3.14	2.29	2.31	2.31	1.94	2.00
25.	I am losing opportunities because I cannot make decisions fast	1.79	1.79	2.07	2.21	1.54	1.62	1.88	1.82
26.	I feel rested*	2.50	2.29	2.36	2.07	2.38	1.77	2.06	1.88
27.	I am calm, cool, and collected*	1.86	1.93	1.64	1.50	1.92	1.62	2.18	1.94
28.	I feel that difficulties are piling up so that I cannot overcome them	1.71	1.57	2.14	1.64	1.85	1.77	1.65	1.47
29.	I worry too much over something that really doesn't matter	1.86	1.71	2.21	1.71	2.46	1.77	2.24	2.18
30.	I am happy*	2.50	2.07	2.07	1.79	2.08	2.00	2.06	1.94
31.	I am inclined to take things hard	1.86	2.07	2.43	2.14	2.00	1.77	2.00	2.12
32.	I lack self-confidence	1.64	1.50	1.71	2.07	1.77	1.85	2.18	1.59
33.	I feel secure*	1.71	1.50	1.57	1.36	1.77	1.69	1.82	1.76
34.	I try to avoid facing a crisis or difficulty	2.36	2.57	2.64	2.43	2.31	2.00	2.00	2.18
35.	I feel blue	2.07	1.79	2.07	1.86	1.69	1.54	1.94	1.88
36.	I am content*	2.50	2.07	2.07	1.86	2.00	1.92	2.06	2.06
37.	Some unimportant thought runs through my mind and bothers me	1.71	1.64	1.64	1.64	1.92	1.77	1.94	1.65
38.	I take disappointments so keenly that I can't put them out of my mind	2.21	1.57	1.93	1.79	1.69	1.62	2.00	2.06
39.	I am a steady person*	1.36	1.50	1.29	1.29	1.54	1.46	1.71	1.35
40.	I become tense and upset when I think about my current concerns	1.86	1.93	2.21	1.43	2.15	1.69	2.12	2.06

Note: Results are mean values. Reverse counting items are shown with "*". The Polish State and Trait Anxiety Inventory is an adaptation of the American State and Trait Anxiety Inventory test developed by C.D. Spielberger, R.L. Gorsuch and R.E. Lushene. The authors of the Polish adaptation of the test are J. Strelau, T. Tysarczyk and K. Wrześniewski. After the standardization and normalization works, the final version of the test was prepared by M. Tysarczyk. Abbreviations: STAI-S, State-Trait Anxiety Inventory – State; STAI-T, State-Trait Anxiety Inventory – Trait; HD, hemodialysis patients; C, patients with normal renal function.
